# LAZY5 acts in an LAZY1‐independent pathway to regulate rice tiller angle

**DOI:** 10.1111/pbi.70211

**Published:** 2025-07-13

**Authors:** Wenguang Wang, Dajun Sang, Peng Li, Songtao Gui, Yan Liang, Yuqi Song, Han Zhang, Jiajia Cao, Zihao Wang, Xiangbing Meng, Hongwei Xue, Jiayang Li, Yonghong Wang

**Affiliations:** ^1^ State Key Laboratory of Wheat Improvement, College of Life Sciences Shandong Agricultural University Tai'an China; ^2^ Yazhouwan National Laboratory Sanya China; ^3^ Shandong Agriculture and Engineering University Jinan China; ^4^ Institute of Genetics and Developmental Biology Chinese Academy of Sciences Beijing China; ^5^ Guangdong Laboratory for Lingnan Modern Agriculture, Guangdong Basic Research Center of Excellence for Precise Breeding of Future Crops, College of Agriculture South China Agricultural University Guangzhou China; ^6^ Shanghai Collaborative Innovation Center of Agri‐Seeds, Joint Center for Single Cell Biology, School of Agriculture and Biology Shanghai Jiao Tong University Shanghai China

**Keywords:** rice, tiller angle, shoot gravitropism, lateral auxin transport, *LAZY5*

## Abstract

Tiller angle is a key agronomic trait that influences plant architecture and thus grain yield by optimizing rice planting density. Although great progress has been made in understanding the *LAZY1*‐dependent pathway mediating rice tiller angle, the genetic regulatory network of rice tiller angle remains to be elucidated. Here, we identified a new tiller angle gene *LAZY5* (*LA5*) that encodes a member of the ATP binding cassette transporter G subfamily (ABCG) transporter. We found LA5 can interact with OsPIN3t to regulate lateral auxin transport (LAT), shoot gravitropism, and thus tiller angle. Further genetic analysis demonstrated that LA5 acts in a novel LA1‐independent pathway to modulate LAT and rice tiller angle. Up‐regulation of *LA5* not only enlarges tiller angle but also increases tiller number in rice. Moreover, *LA5* was strongly selected during rice domestication, with haplotype differentiations happened within the *indica* rice population. Our study not only uncovers a novel *LA1*‐independent pathway controlling LAT and shoot gravitropism, but also provides a potential molecular target for high‐yield breeding via synergistically regulating rice tiller angle and tiller number.

## Introduction

Rice (*Oryza sativa* L.) tiller angle is defined as the angle between the vertical line and the side tillers with maximum inclination, affecting rice planting density and photosynthesis efficiency (Wang *et al*., 2022). In practice, the extremely spread or compact plant architecture exerts adverse effects on rice production, and appropriate tiller angle is crucial for the ideal plant architecture and thus grain yield of rice (Gao *et al*., 2019; Wang *et al*., 2022; Wang and Li, 2008; Xu *et al*., 1998). Therefore, elucidating the molecular mechanisms underlying rice tiller angle would substantially contribute to high‐yield breeding goals in rice.

The growth habit transition from the prostrate wild rice to the erect cultivated rice has greatly increased rice grain yield. Several genes, including *PROSTRATE GROWTH1* (*PROG1*), *PROG7* and *RICE PLANT ARCHITECTURE DOMESTICATION* (*RPAD*), have been found to be involved in the prostrate‐to‐erect growth habit transition during rice domestication (Hu *et al*., 2018; Jin *et al*., 2008; Tan *et al*., 2008; Wu *et al*., 2018). Asian cultivated rice is composed of two major subspecies, *O. sativa* ssp. *japonica* and *indica*, which show significantly different tiller angles (Wang *et al*., 2022; Xu and Sun, 2021). Some genes, such as *Tiller Angle Control 1* (*TAC1*), *TAC3*, *TAC4*, *TILLER INCLINED GROWTH 1* (*TIG1*) and *DWARF2* (*D2*), have been identified as key regulators responsible for the variation of tiller angles between *japonica* and *indica* rice (Dong *et al*., 2016; Jiang *et al*., 2012; Li *et al*., 2021; Wang *et al*., 2022; Yu *et al*., 2007; Zhang *et al*., 2019).

Previous studies have revealed that shoot gravitropism largely contributes to the establishment of rice tiller angle (Gao *et al*., 2019; He *et al*., 2021; Wang *et al*., 2022). The gravity‐directed growth process, called gravitropism, directs roots downwards (positive gravitropism) and shoots upwards (negative gravitropism), respectively (Chen *et al*., 1999). Positive/negative gravitropism ensures that roots take up the water and minerals from soil, and shoots grow in the air for gas exchange and efficient photosynthesis, respectively (Morita, 2010). Shoot gravitropism enables crops to re‐grow upward in response to logging caused by rainstorm, reducing yield lost and facilitating mechanized harvesting. Thus, shoot gravitropism plays a crucial role in crop production.

In higher plants, gravitropism is traditionally divided into the following sequential steps: gravity sensing, gravity signal transduction, auxin redistribution and growth response (Fukaki *et al*., 1996; Strohm *et al*., 2012). Over the past decades, some key genes functioning in different steps of shoot gravitropism have been identified as regulators of rice tiller angle (Wang *et al*., 2022). Several genes, such as *AGPL1*, *AGPL3*, *LAZY2* (*LA2*), *OspPGM* and *LA3*, act at the gravity sensing step by controlling starch levels in gravity‐sensing tissues to regulate shoot gravitropism and tiller angle in rice (Cai *et al*., 2023; Huang *et al*., 2021; Okamura *et al*., 2013, 2015). Recent studies found that *LA4*/ *LATA1* acts additively with the starch–statolith‐dependent gravity‐sensing pathway to regulate shoot gravitropism (Fan *et al*., 2024; Wang *et al*., 2024). In addition, *Loose Plant Architecture1* (*LPA1*) and *ONAC106* regulate shoot gravity perception by controlling the amyloplast sedimentation rate (Sakuraba *et al*., 2015; Wu *et al*., 2013).

Lateral auxin transport (LAT) also plays a crucial role in controlling shoot gravitropism and plant architecture (Wang *et al*., 2022). The *Arabidopsis* auxin efflux transporter PIN3 was firstly reported to control lateral auxin transport to regulate shoot gravitropism (Friml *et al*., 2002; Rakusova *et al*., 2011). Among the 12 putative rice auxin efflux carrier homologues of AtPIN proteins, OsPIN3t shows the highest sequence identity to AtPIN3 and was found to regulate drought stress response (Zhang *et al*., 2012). However, the detailed molecular function of OsPIN3t is still unclear in rice. In recent years, some genes have been reported to regulate lateral auxin transport, gravitropic response and tiller angle, acting in the LA1‐dependent pathway in rice (Harmoko *et al*., 2016; Hu *et al*., 2020; Li *et al*., 2007, 2019, 2020; Wang *et al*., 2023b; Yoshihara and Iino, 2007; Zhang *et al*., 2018, 2023; Zhao *et al*., 2015; Zhu *et al*., 2020). The first identified tiller angle gene *LA1* encodes a novel grass‐specific protein, regulating shoot gravitropism and tiller angle by affecting lateral auxin transport, and the knockout mutant of *LA1* showed altered endogenous IAA distribution and substantially reduced shoot gravitropism (Li *et al*., 2007; Yoshihara and Iino, 2007). It was demonstrated that the nuclear localization of LA1 is regulated by its interacting protein BRXL4 (Li *et al*., 2019). The transcriptional level of *LA1* is repressed by PROG1; meanwhile, LA1 can also inhibit the expression of *PROG1* by directly binding to its promoter (Wang *et al*., 2023a,b; Zhang *et al*., 2023). Besides, there are multiple transcriptional regulators reported to control rice tiller angle by acting through the *LA1*‐dependent pathway, including HSFA2D, WOX6/11, OsHOX1/28, OsARF5 and OsARF12/17/25 (Hu *et al*., 2020, 2025; Li *et al*., 2020; Zhang *et al*., 2018). It is worth noting that the loss‐of‐function mutant *la1* only showed partially impaired shoot gravitropism, implying that an unidentified *LA1*‐independent signalling pathway is also involved in the regulation of rice shoot gravitropism (Yoshihara and Iino, 2007). However, few pieces of evidence have been documented in terms of the *LA1*‐independent shoot gravitropism regulatory pathway.

In this study, we identified a new rice tiller angle gene *LA5.* We found that *LA5* encodes a member of the ATP binding cassette transporter G subfamily (ABCG) that can interact with auxin transporter OsPIN3t to regulate lateral auxin transport, shoot gravitropism, and thus tiller angle in rice. We further demonstrated that *LA5* acts in a novel *LA1*‐independent gravity signalling pathway to control lateral auxin transport and rice tiller angle, providing novel insights into the rice tiller angle regulatory network.

## Results

### Phenotypic characterization of the tiller angle mutant *la5‐Dominant* (*la5‐D*) in rice

**Figure 1:**
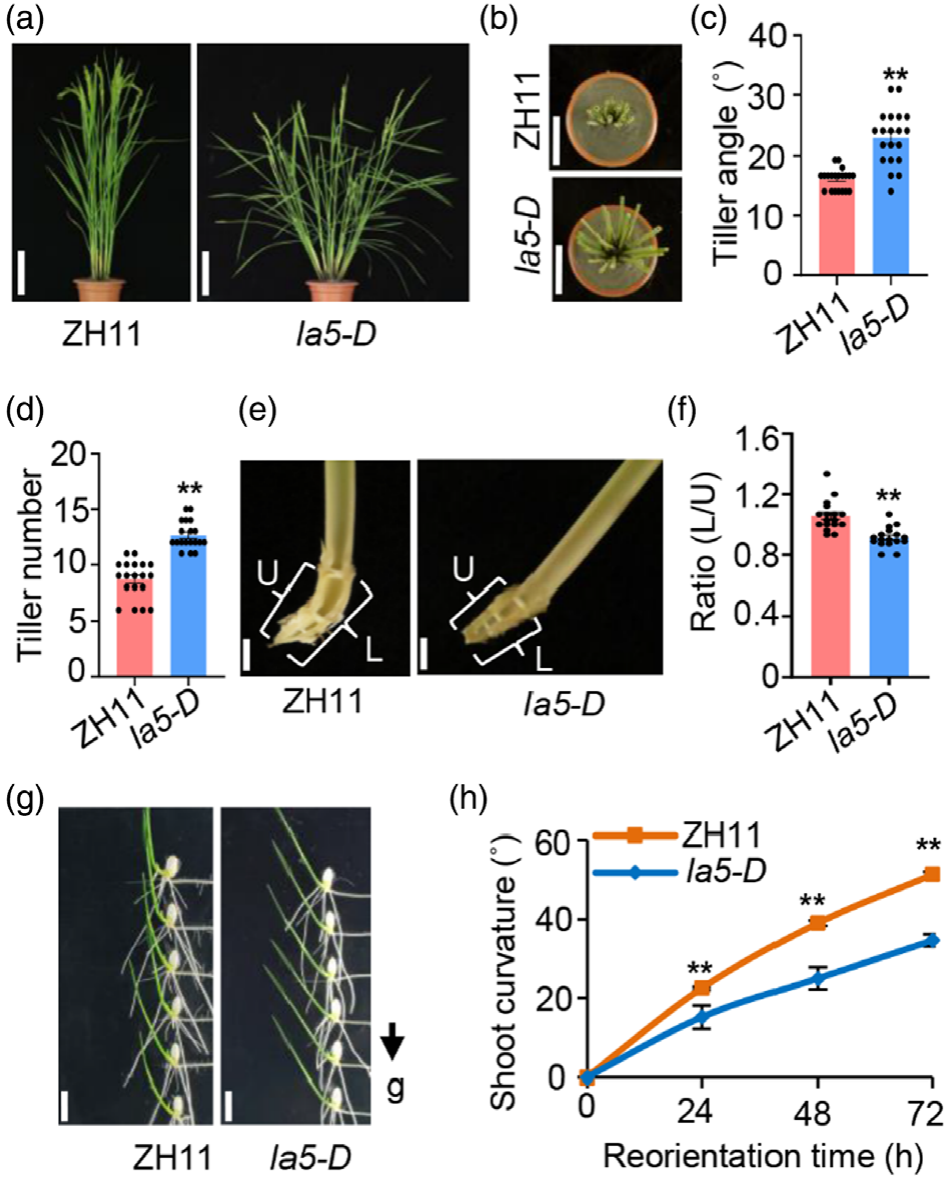
Phenotypic characterization of the rice mutant *la5‐D*. (a) The gross morphologies of the wild‐type ZH11 and *la5‐D* at the adult stage. Scale bars, 25 cm. (b) Comparison of the tiller bases between ZH11 and *la5‐D* at the adult stage. Scale bars, 10 cm. (c) Statistical analysis of the tiller angle of ZH11 and *la5‐D* at the adult stage. Data are presented as mean ± SE (*n* = 20). **P < 0.01, Student's *t*‐test. (d) Statistical analysis of the tiller number of ZH11 and *la5‐D* at the adult stage. Data are presented as mean ± SE (*n* = 20). **P < 0.01, Student's *t*‐test. (e) Asymmetric growth of tiller bases of ZH11 and *la5‐D*. Lower side (L), upper side (U). Scale bars, 1 cm. (f) The ratio of the length between the lower side and upper side in ZH11 and *la5‐D* at the adult stage. Data are presented as mean ± SE (*n* = 15). **P < 0.01, Student's *t*‐test. (g) Gravitropism assay for *la5‐D* under the light after gravistimulation for 72 h. Scale bars, 1 cm. The black arrow indicates the direction of gravity. (h) Statistical analysis of shoot curvature grown under the light after gravistimulation. Data are presented as mean ± SE (*n* = 18). **P < 0.01, Student's *t*‐test.

To elucidate the molecular mechanism underlying rice tiller angle, we isolated a dominant rice mutant with loose plant architecture, designated as *la5‐D*, from the mutagenesis library in the *japonica* rice variety ZH11 (wild type) background. Phenotypic observation and statistical analysis showed that not only the tiller angle was enlarged but also the tiller number was increased in *la5‐D* compared with that in ZH11 (Figure 1a–d). Further observation of a longitudinal section of the tiller base and statistical analysis showed that the tiller base length ratio of the near‐ground side to the far‐ground side decreased significantly in *la5‐D* compared with that in ZH11 (Figure 1e,f). Shoot gravitropism plays a predominant role in the formation of rice tiller angle (Wang *et al*., 2022). To verify the involvement of *LA5* in regulating shoot gravitropism, we examined the gravitropic response of coleoptiles grown in the dark and found that the coleoptiles of *la5‐D* responded more slowly than ZH11 after gravity stimulation for 4 h (Figure S1a,b). The gravitropic responses of young seedlings and adults were also tested. After being placed horizontally, the gravitropic response of *la5‐D* was significantly reduced compared with ZH11 at both stages (Figure 1g,h, Figure S1c,d for young seedlings; Figure S1e,f for adults). These results demonstrated that *LA5* negatively regulates shoot gravitropism, and the defective shoot gravitropism accounts for the larger tiller angle of the *la5‐D* mutant.

### *LA5* negatively regulates rice polar auxin transport (PAT) and lateral auxin transport (LAT)

**Figure 2:**
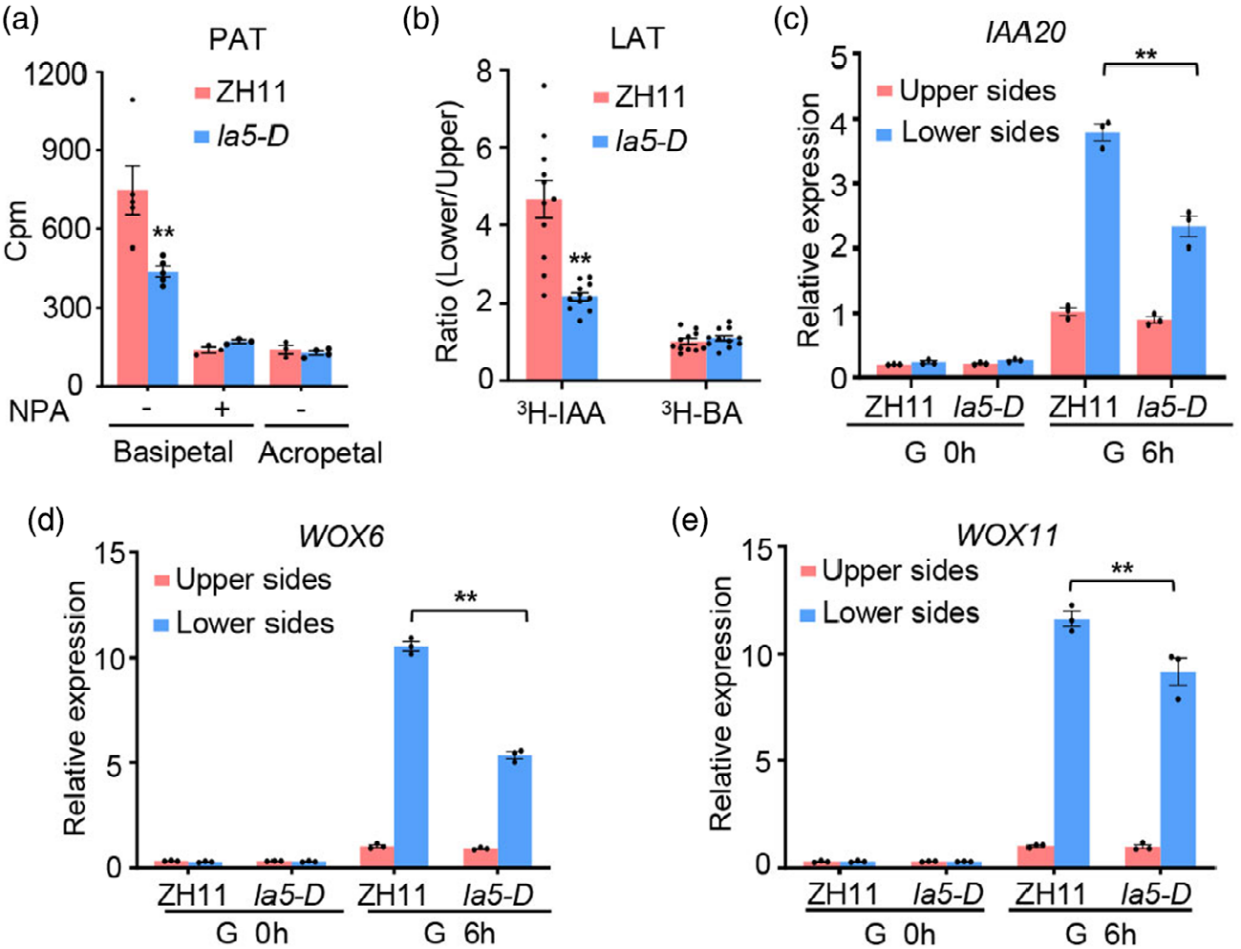
*LA5* regulates asymmetric auxin distribution upon gravistimulation. (a) Polar ^3^H‐IAA transport in coleoptiles of ZH11 and *la5‐D*. +/− means with/without NPA treatment. Data are presented as mean ± SE (*n* = 5). **P < 0.01, Student's *t*‐test. (b) Lateral ^3^H‐IAA transport in coleoptiles of ZH11 and *la5‐D*. The Cpm ratio represents the radioactivity ratio between the lower side and the upper side of coleoptiles upon gravity stimulation. Data are presented as mean ± SE (*n* = 11). **P < 0.01, Student's *t*‐test. (c–e) Expression levels of *IAA20* (c), *WOX6* (d), and *WOX11* (e) in the lower side and the upper side of the shoot bases upon gravistimulation for 0 h and 6 h, respectively. Data are presented as mean ± SE (*n* = 3). **P < 0.01, Student's *t*‐test.

To further investigate whether the defective shoot gravitropism of *la5‐D* results from alteration of PAT and LAT as those in *la1*, we checked the PAT and LAT in etiolated coleoptiles of *la5‐D* plants. We applied ^3^H‐labelled indole acetic acid (IAA) to the apical side of etiolated coleoptiles and measured the amount transported to the basal end. Results revealed that the basipetal PAT in *la5‐D* was reduced significantly compared with that in ZH11, and PAT in both WT and *la5‐D* was almost completely blocked when treated with the auxin efflux transporter inhibitor napthylpthalamic acid (NPA) (Figure 2a). As a control, acropetal PAT showed no significant differences between *la5‐D* and ZH11 (Figure 2a). We further measured the lateral ^3^H‐IAA transport in coleoptiles of *la5‐D* upon gravity stimulation. Results showed that the radioactivity ratio of the lower halves to upper halves of *la5‐D* was significantly lower than that in ZH11, revealing that LAT was greatly reduced in *la5‐D* and consequently asymmetric IAA distribution was impaired in *la5‐D* upon gravity stimulation (Figure 2b). As a control compound, ^3^H‐benzoic acid (^3^H‐BA) displayed no difference between *la5‐D* and ZH11 in the radioactivity ratio of the lower to upper halves (Figure 2b). To further verify that asymmetric IAA distribution is impaired in *la5‐D* seedlings upon gravistimulation, we also checked the transcription levels of auxin responsive marker genes *IAA20*, *WOX6* and *WOX11* in the shoot bases upon gravistimulation. After gravity stimulation for 6 h, the induced expressions of *IAA20*, *WOX6*, and *WOX11* were all significantly lower in the lower sides of *la5‐D* compared with ZH11 (Figure 2c–e). These results further demonstrated that asymmetric IAA distribution was impaired in *la5‐D* upon gravistimulation. Taken together, our findings demonstrated that the larger tiller angle and attenuated shoot gravitropism of *la5‐D* were caused by the defective asymmetric auxin distribution upon gravistimulation.

### Cloning and functional confirmation of the *LA5* gene

**Figure 3:**
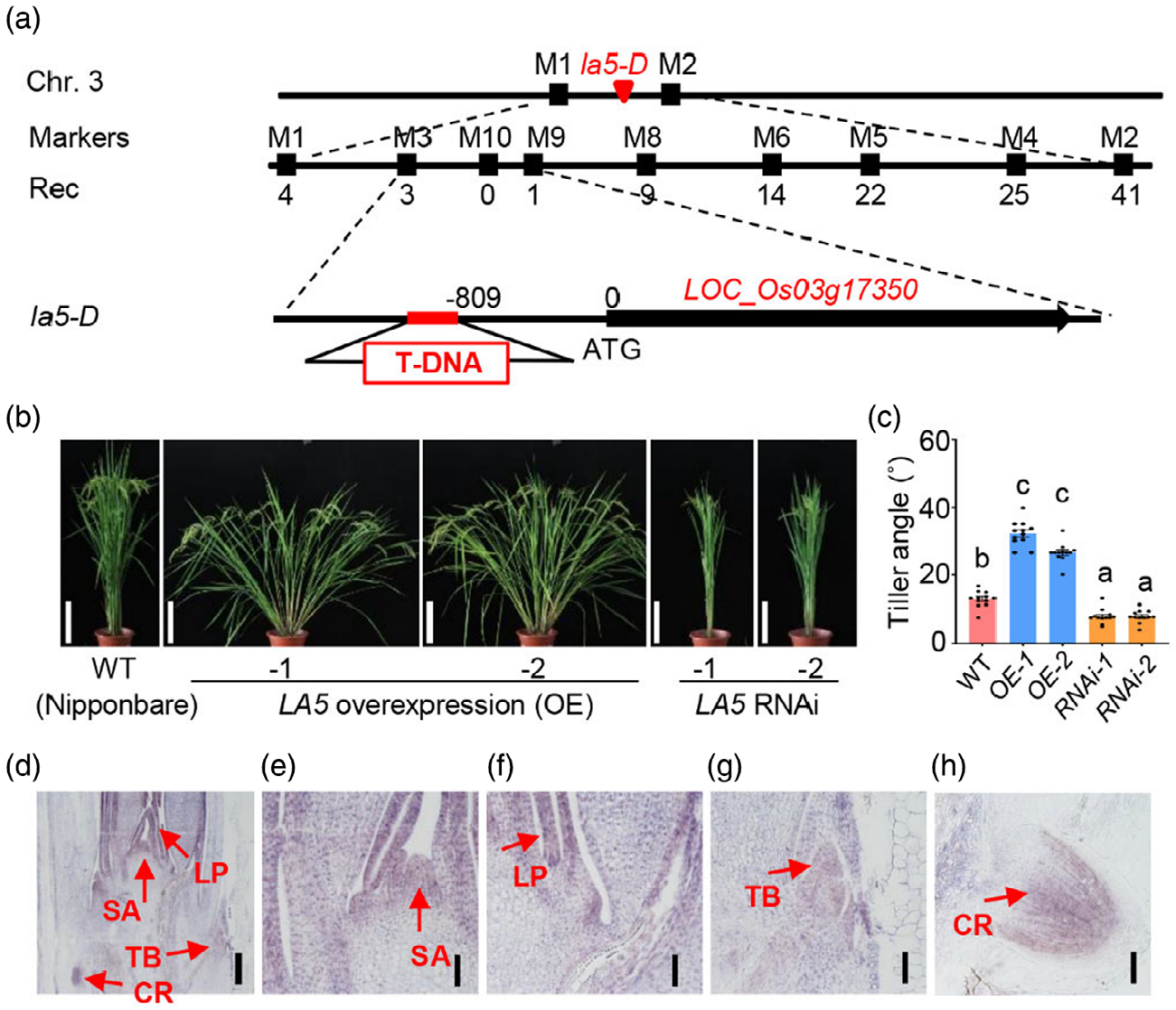
Map‐based cloning and functional confirmation of *LA5*. (a) *LA5* was roughly mapped on chromosome 3, linked to Indel marker M1 and marker M2. *LA5* was further narrowed down to a 213‐kb interval between marker M3 and M9. A T‐DNA insertion was identified in the promoter of *LOC_Os03g17350*. (b) Phenotypes of overexpressing and RNAi transgenic plants of *LA5*. Scale bars, 25 cm. (c) Statistical analysis of the tiller angle of the overexpressing and RNAi transgenic plants of *LA5*. Data are presented as mean ± SE (*n* = 10). Different letters above the column represent a statistically significant difference at *P* < 0.05 (one‐way ANOVA, Tukey's honestly significant difference). (d) *In situ* of longitudinal section of the shoot apex of young seedlings. Scale bar, 200 μm. CR, lateral root primordium; LP, leaf primordium; SA, shoot apical meristem; TB, tiller bud. (e–h) Partial enlarged views of longitudinal section of the shoot apical meristem in young seedlings. (e–g), Scale bars, 50 μm; (h), Scale bar, 100 μm.

To isolate the *LA5* gene, we generated a F_2_ population derived from the cross between *la5‐D* and 93–11 (a wild‐type *indica* variety). The gene was narrowed down to a 213‐kb region between markers M3 and M9 (Figure 3a). Using RT‐PCR to examine transcriptional levels of candidate genes in the region, we found that the expression of *LOC_Os03g17350* was significantly higher in *la5‐D* compared with ZH11 (Figure S2a), which was caused by a T‐DNA insertion in the promoter position (−809 bp) of *LOC_Os03g17350* as revealed by sequencing analysis (Figure 3a and Figure S2b,c). *LOC_Os03g17350* encodes an ATP binding cassette (ABC) transporter of a G subfamily protein OsABCG5/RCN1, which functions pleiotropically including the regulation of tiller number, plant height, leaf number, panicle morphology, root architecture and stomatal closure in rice (Ariyaratna *et al*., 2011; Lee *et al*., 2011; Matsuda *et al*., 2016; Shiono *et al*., 2014; Yasuno *et al*., 2007, 2009).

To validate the tiller‐spreading effects of *LOC_Os03g17350*, we generated its overexpression (*LA5* OE) and RNAi transgenic lines (*LA5* RNAi) in the *japonica* rice variety Nipponbare background. The RT‐qPCR analysis showed that the transcriptional level of *LOC_Os03g17350* was significantly up‐regulated in *LA5* OE lines while decreased in the *LA5* RNAi transgenic lines, respectively (Figure S3a). All 10 independent *LA5* OE lines exhibited larger rice tiller angle (Figure 3b,c) while the 12 independent *LA5* RNAi transgenic lines displayed more erect plant architecture (Figure 3b,c). We also checked the shoot gravitropism of *LA5* OE and *LA5* RNAi transgenic plants and found that the shoot gravitropism was significantly reduced in *LA5* OE plants while enhanced in the *LA5* RNAi plants (Figure S3b,c), further demonstrating that *LA5* negatively regulates shoot gravitropism in rice. In addition to the alteration of rice tiller angle, we noticed that the tiller number is also changed in these transgenic lines (Figure 3b). The tiller number was significantly increased in the *LA5* OE lines while decreased in the *LA5 RNAi* lines (Figure S3d), which is consistent with reduced tillering phenotype of *rcn1*. To further confirm the function of *LA5* in regulating tiller angle and tiller number, we also generated its loss‐of‐function mutant lines in *japonica* rice variety ZH11 by using CRISPR‐Cas9 (CR) gene editing. Using two single guide RNA (sgRNA) targeting at different regions of the exons, respectively, we generated two independent loss‐of‐function homozygous mutant *CR‐la5* transgenic lines (Figure S4a). In contrast to the spread‐out phenotype of *LA5* OE lines, the loss‐of‐function mutants showed erect growth and greatly reduced tiller number (Figure S4b). These results strongly confirmed that *LOC_Os03g17350* was the *LA5* gene, which not only positively controls tiller number but also negatively regulates shoot gravitropism to increase tiller angle in rice.

### Expression pattern of *LA5* revealed by RT‐qPCR and mRNA *in situ* hybridization

To investigate the gene expression patterns of *LA5*, we performed RT‐qPCR with RNA samples from various tissues. As shown in Figure S5, *LA5* was expressed ubiquitously in the examined organs/tissues, showing high expression levels in roots, seedlings and shoot bases. The tissue‐specific expression pattern of *LA5* was further examined by mRNA *in situ* hybridization in longitudinal sections through seedling apexes. It was shown that *LA5* is predominantly expressed in tiller buds, shoot apical meristems, leaf primordiums and lateral root primordiums (Figure 3d–h). The high expressions of *LA5* in shoot bases and tiller buds are consistent with the expected role of *LA5* in regulating tiller angle and tiller number.

### *LA5* regulates lateral auxin transport and rice tiller angle in a novel *LA1* ‐independent pathway

**Figure 4:**
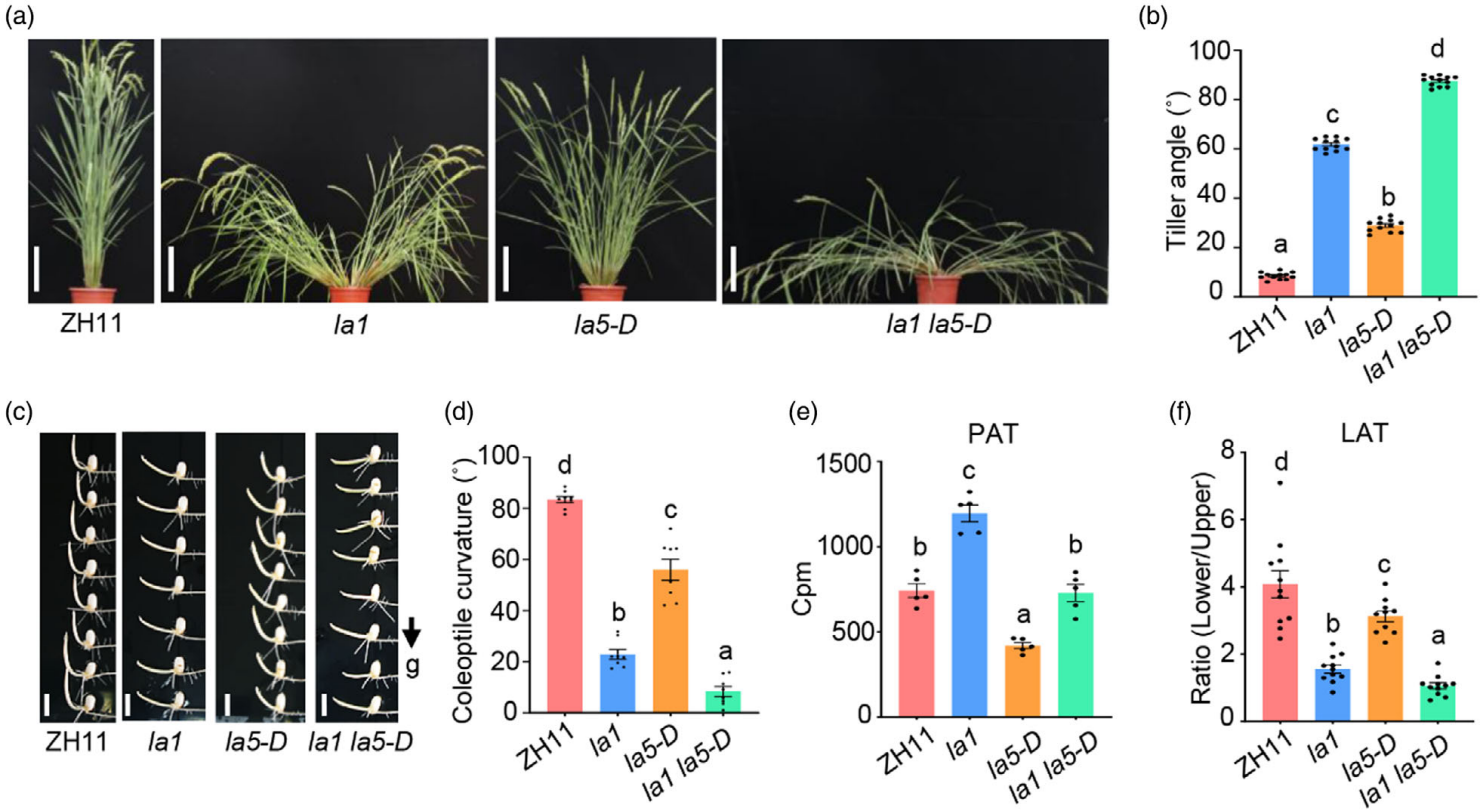
*LA5* acts in the *LA1*‐independent pathway controlling rice tiller angle and shoot gravitropism. (a) Phenotype of the *la1 la5‐D* mutant. Scale bars, 25 cm. (b) Statistical analysis of the tiller angle of the *la1 la5‐D* mutant. Data are represented as mean ± SE (*n* = 12). Different letters above the column represent statistically significant differences at *P* < 0.05 (one‐way ANOVA. Tukey's honestly significant difference). (c) Coleoptiles gravitropism of *la1 la5‐D* mutant. g, gravity. Black arrow indicates the direction of gravity. Scale bars, 1 cm. (d) Statistical analysis of the coleoptile curvature angle of the *la1 la5‐D* mutant. Data are represented as mean ± SE (*n* = 12). Different letters above the column represent statistically significant different at *P* < 0.05 (one‐way ANOVA. Tukey's honestly significant difference). (e) Polar ^3^H‐IAA transport in coleoptiles of the *la1 la5‐D* mutant. Data are represented as mean ± SE (*n* = 5). Different letters above the column represent statistically significant differences at *P* < 0.05 (one‐way ANOVA. Tukey's honestly significant difference). (f) Lateral ^3^H‐IAA transport in coleoptiles of *la1 la5‐D* mutant. The Cpm ratio represents the radioactivity ratio between the lower side and the upper side of coleoptiles upon gravity stimulation. Data are represented as mean ± SE (*n* = 11). Different letters above the column represent statistically significant different at *P* < 0.05 (one‐way ANOVA. Tukey's honestly significant difference).

*LA1* is involved in auxin transport and redistribution upon gravistimulation and plays important roles in regulating rice shoot gravitropism and tiller angle (Li *et al*., 2007; Yoshihara and Iino, 2007). To test whether *LA5* functions in the *LA1*‐dependent pathway, we crossed the *la1* mutant with *la5‐D* to generate the *la1 la5‐D* double mutant. Phenotypic analysis revealed that the *la1 la5‐D* plant showed significantly larger tiller angle than single mutants either (Figure 4a,b). In addition, the shoot gravitropic response of both the coleoptiles and seedlings in the *la1 la5‐D* plant was much slower than single mutants either (Figure 4c,d; Figure S6a,b). Remarkably, the shoots of the *la1 la5‐D* plant grew along the ground and exhibited almost complete loss of shoot gravitropism (Figure 4a–d and Figure S6). These results implied that *LA5* could still exert its function in the absence of *LA1*, indicating that *LA5* acts in a different pathway from *LA1* in the control of shoot gravitropism and rice tiller angle. Previous studies found that *la1* showed reduced LAT and enhanced PAT, and the *LA1*‐dependent pathway involved asymmetric distribution of auxin between the two lateral halves (Li *et al*., 2007; Yoshihara and Iino, 2007). We measured the PAT in *la1 la5‐D* and found that *la5‐D* could reduce the PAT of *la1*, and *la1 la5‐D* showed similar PAT to the ZH11 (Figure 4e), implying that reduced PAT in *la5‐D* was not responsible for its larger tiller angle. Strikingly, LAT analysis showed that *la1 la5‐D* exhibited almost completely abolished LAT, which was only partially defective in both single mutants (Figure 4f). These results revealed that *LA5* regulates shoot gravitropism and tiller angle by acting in a novel *LA1*‐independent pathway to modulate lateral auxin transport.

### LA5 interacts with OsPIN3t to regulate shoot gravitropism and tiller angle in rice

**Figure 5:**
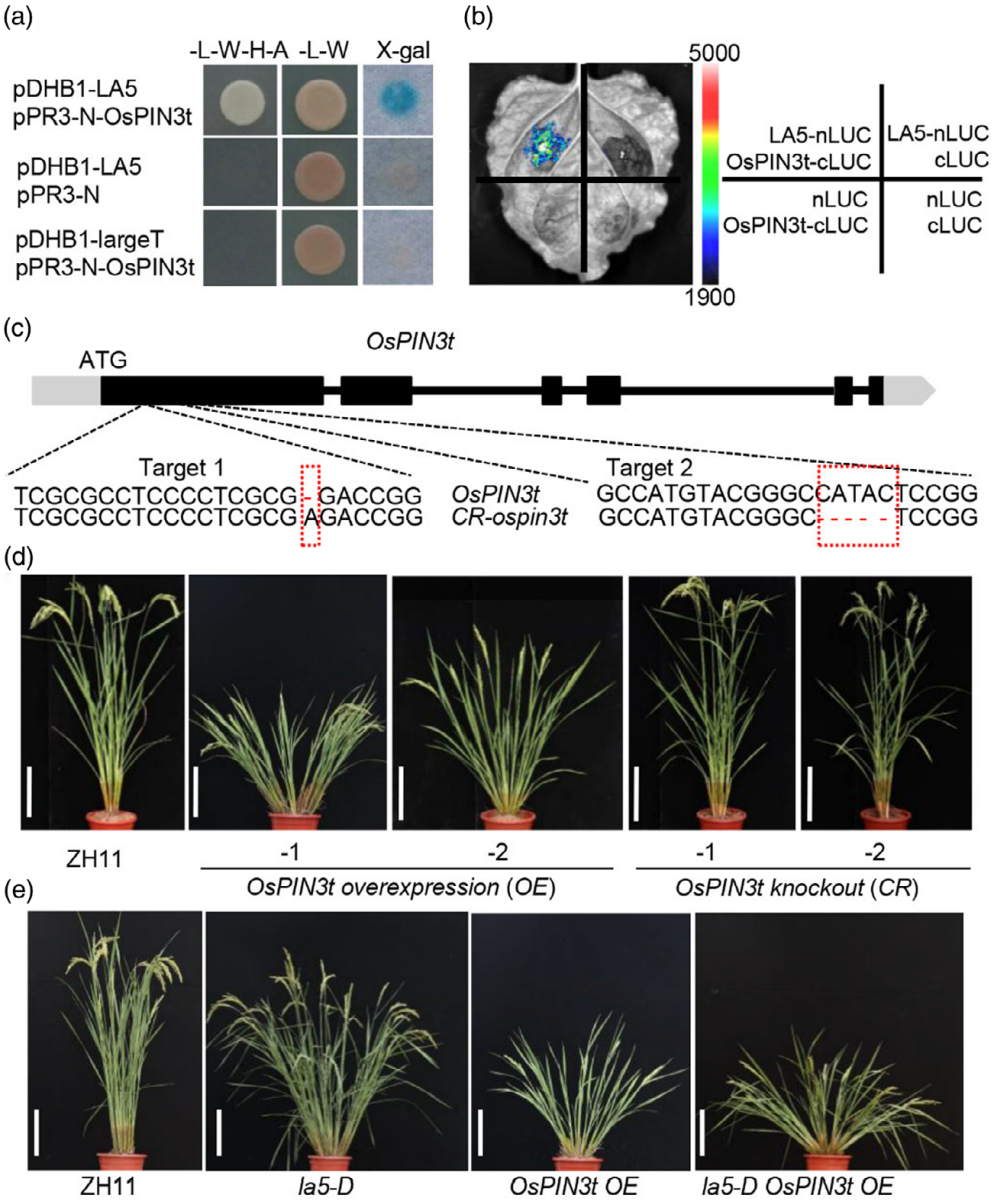
LA5 interacts with OsPIN3t to regulate rice tiller angle. (a) LA5 interacts with OsPIN3t in yeast. ‐L‐W‐H‐A denotes the medium lacking Leu, Trp, His and Ade. ‐L‐W denotes the medium lacking Leu, Trp. Blue colour in an X‐Gal assay indicates the two proteins can interact in this system. (b) LA5 interacts with OsPIN3t in LUC assay. The pseudocolour bar showed the luminescence intensity in the image. (c) Schematics illustrating and sequence alignment of the two gRNA targeting sites of *OsPIN3t* gene in the CRISPR‐Cas9 (CR) engineered *CR‐ospin3t* mutant lines. Red box indicated the mutation positions. (d) Gross morphologies of the ZH11, *OsPIN3t OE* (1 and 2) and *CR‐ospin3t* (1 and 2). Scale bars, 20 cm. (e) Gross morphologies of the ZH11, *la5‐D*, *OsPIN3t OE* and *la5‐D OsPIN3t OE* at the adult stage. Scale bars, 20 cm.

To further elucidate the molecular mechanism underlying LA5‐mediated LAT and rice tiller angle, we performed a yeast two‐hybrid assay to screen for the LA5‐interacting proteins. Among the candidate interactors, the auxin transport protein OsPIN3t showed interaction with LA5 in yeast (Figure 5a, top panel). To verify the interaction between LA5 and OsPIN3t, we further carried out a luciferase complementation imaging (LCI) assay. As shown in Figure 5b (upper left panel), LA5 can interact with OsPIN3t.

In order to confirm whether *OsPIN3t* is also involved in controlling rice tiller angle, we generated its loss‐of‐function mutant lines in ZH11 by using CRISPR‐Cas9 (CR) gene editing. Using two single guide RNAs (sgRNAs) targeting different regions of the exon respectively, we generated two independent loss‐of‐function homologous mutant transgenic lines (Figure 5c), and the CR‐engineered *OsPIN3t* (*CR‐ospin3t*) mutant lines showed erect plant architecture (Figure 5d). We also got the overexpression transgenic plants of *OsPIN3t*, and the increased expression of *OsPIN3t* caused an larger tiller angle (Figure S7a and Figure 5d). To further test whether asymmetric IAA distribution is impaired in *OsPIN3t OE* upon gravistimulation, we checked the transcript levels of auxin responsive marker genes *IAA20*, *WOX6* and *WOX11* in the shoot bases upon gravistimulation. After gravity stimulation for 6 h, *IAA20, WOX6* and *WOX11* were all expressed asymmetrically in both *OsPIN3t OE* and ZH11, but their induced expressions were significantly lower in the lower sides of the shoot bases of *OsPIN3t OE* than the counterparts in ZH11 (Figure S7b–d), implying defective asymmetric auxin distribution in the *OsPIN3t OE* plants upon gravistimulation. These results demonstrated that *OsPIN3t* positively regulates tiller angle in rice.

In addition, we also generated the *la5‐D OsPIN3t OE* plants by crossing the *la5‐D* with *OsPIN3t OE‐1*. The gross morphologies of *la5‐D OsPIN3t OE‐1* plants were more similar to the *OsPIN3t OE‐1* plants (Figure 5e). Together, these results suggested that LA5 could interact with OsPIN3t to regulate shoot gravitropism and tiller angle by affecting lateral auxin transport in rice.

### Haplotypes and nucleotide diversity analysis of *LA5* in rice

**Figure 6:**
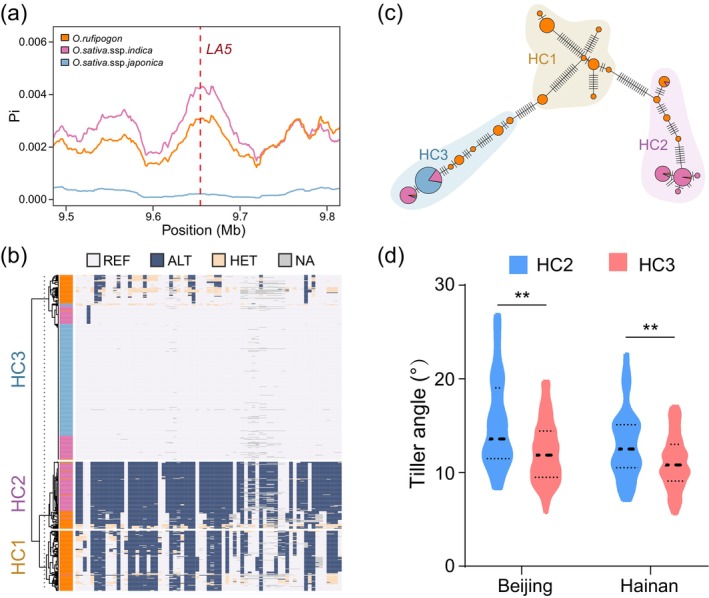
Haplotype and selection analysis of *LA5*. (a) Distribution of nucleotide diversity of *LA5* among *O. sativa* ssp. *japonica*, *O. sativa* ssp. *indica* and *O. rufipogon* populations. Red dashed line indicated the position of *LA5*. (b) Haplotype cluster of *LA5*. The group colour on the left of the heatmap indicated the species of each sample, with the same colour rules as those in (a). HC1~HC3 indicated different haplotype clusters of *O. rufipogon*, *O. sativa*. ssp. *indica* and *O. sativa*. ssp. *japonica* populations, respectively. (c) Haplotype network of *LA5*. Each node indicated the proportion of different species with the same classification and colour rules as those in (a) in a haplotype, the segments on each edge indicated the edit distance between two nearest haplotypes, HC1~HC3 indicated the haplotype clusters in (b). (d) Tiller angle of HC2 and HC3 of *LA5* of rice grown in Beijing and Hainan, respectively. *n* = 47~256. **P < 0.01, Student's *t*‐test.

In order to study the natural variation of *LA5*, we analysed the nucleotide diversity of *LA5* and found that the nucleotide diversity of *LA5* in *japonica* rice populations was significantly lower than that in *indica* and wild rice populations, indicating selection of *LA5* in *japonica* rice (Figure 6a). Haplotype analysis showed that the haplotypes of *LA5* can be grouped into three haplotype clusters (HCs). While HC1 seems to be the oldest cluster for containing only the *O. rufipogon* accessions, the *indica* rice accessions appeared in both HC2 and HC3, and *japonica* rice accessions were only found in HC3 (Figure 6b). Further haplotype network analysis revealed that HC2 and HC3 were distant from each other, separated by HC1 as the intermediate type (Figure 6c), indicating that both the HC2 and HC3 of *LA5* were selected and differentiated during rice domestication. The geographical distribution showed that, when compared to the HC1 accessions, HC3 accessions distributed in higher latitude regions while HC2 accessions were distributed in lower latitude regions (Figure S8). We compared the tiller angle between HC2 and HC3 haplotypes and found that the tiller angle of HC2 was larger than that in HC3 (Figure 6d). These results indicated that *LA5* was strongly selected and differentiated during rice domestication, which contributed to the tiller angle variation in different rice‐distributed regions. Besides, the occurrence of haplotype differentiation within the *indica* rice population implied that either *LA5* was selected according to pressures other than the *indica–japonica* differentiation in rice, or *LA5* was selected during *indica–japonica* differentiation followed by gene exchanges between some *indica* rice and *japonica* rice in East Asia and South Asia.

## Discussion

**Figure 7:**
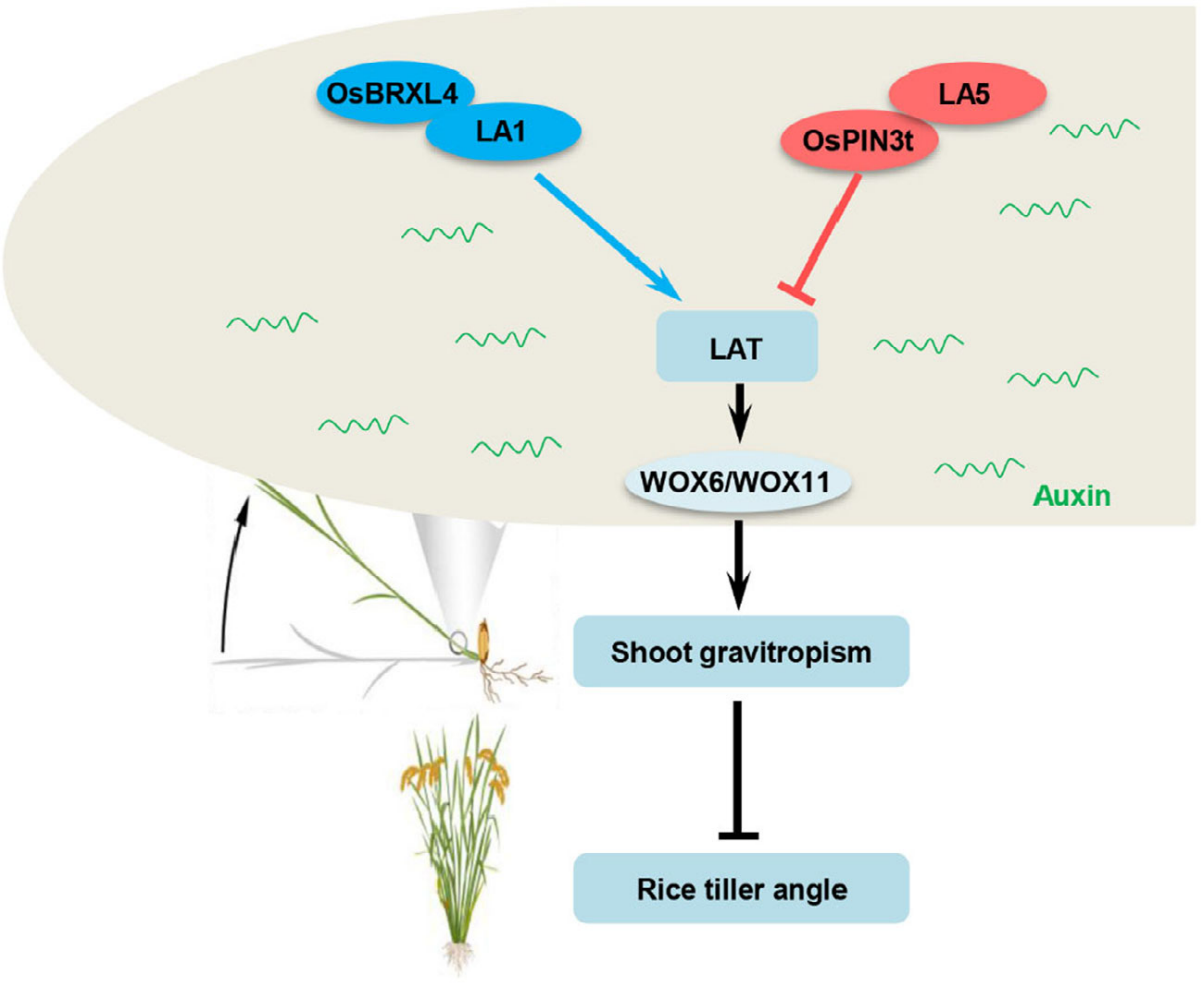
A proposed working model of *LA5*‐mediated *LA1*‐independent asymmetric auxin distribution. LA5 could interact with OsPIN3t to regulate shoot gravitropism and rice tiller angle, acting in a novel LA1‐independent gravity signalling pathway. LA5 negatively regulates LAT while LA1 can interact with OsBRXL4 to positively regulate LAT. By negatively regulating LAT, LA5 modulates auxin asymmetric distribution, which in turn induces the asymmetric expression of auxin downstream regulators *WOX6* and *WOX11*, consequently regulating shoot gravitropism and rice tiller angle. LAT, lateral auxin transport.

Tiller angle is an important agronomic trait that contributes to crop yields by determining the plant density in crop production (Wang and Li, 2005) Although significant progress has been achieved in characterizing tiller angle mutants and corresponding regulatory genes, the molecular mechanisms underlying tiller angle control remain to be elucidated in rice. Rice tiller angle is also an ideal system for understanding the mechanism of shoot gravitropism. In this study, we proposed a novel *LA1*‐independent tiller angle regulatory pathway modulating lateral auxin transport, thus determining rice shoot gravitropism (Figure 7). In this pathway, the novel tiller angle regulator LA5 modulates auxin asymmetric distribution via interaction with OsPIN3t acting in the auxin transport. *LA5* and *LA1* are two major genes controlling lateral auxin transport but act in a different manner to regulate shoot gravitropism and tiller angle. Our study sheds light on a novel molecular mechanism of *LA5* involved in shoot gravitropism and tiller angle in an *LA1*‐independent way in rice.

### *LA5* acts as a pleiotropic regulator of rice plant architecture

Rice plant architecture, a collection of the important agronomic traits including tiller number, tiller angle, plant height and panicle morphology, determines grain yield (Wang and Li, 2005). Previous studies found that *OsABCG5/RCN1* regulated tiller number, plant height, leaf and panicle morphology, root architecture and stomatal closure in rice (Ariyaratna *et al*., 2011; Lee *et al*., 2011; Matsuda *et al*., 2016; Shiono *et al*., 2014; Yasuno *et al*., 2007, 2009). In our study, down‐regulation or knockout of *LA5* resulted in less tiller number, which is consistent with the *rcn1‐1* and *rcn1‐2* mutants with amino acid substitution (Yasuno *et al*., 2009). In addition, we also found overexpression of *LA5/RCN1/OsABCG5* can significantly increase the tiller number (Figure S3d), similar to some PAT‐defective mutants with increasing branch numbers such as *bushy and dwarf 1* (*bud1*) in *Arabidopsis* (Dai *et al*., 2006). More importantly, we also demonstrated the novel function of *LA5* to regulate rice tiller angle by controlling shoot gravitropism and lateral auxin transport in this study (Figures 1 and 2b–e and Figure S1). It is likely that *LA5* controls tiller number through influencing PAT, while it regulates shoot gravitropism and tiller angle through modulating LAT. As a master regulator of rice plant architecture, *LA5* is a promising molecular target for high‐yield breeding through balancing rice tiller angle and tiller number.

Plant ABC transporters consist of the largest family members among many other membrane transporters and play various functions such as disease resistance, detoxification and transport of different substrates (Yang *et al*., 2013). The ABCG half‐transporters must dimerize to form a full, functional ABC transporter as homodimers or heterodimers (McFarlane *et al*., 2010). In *Arabidopsis thaliana*, ABCG11 not only can form heterodimers with ABCG12 but also can form homodimers, and the mutant *abcg11* showed a pleiotropic phenotype (McFarlane *et al*., 2010). Previous studies showed that RCN1/OsABCG5 can homodimerize and heterodimerize with OsABCG2 and OsABCG3 (Matsuda *et al*., 2016). Different combinations of ABCG transporters can perform distinct functions. Moreover, ABCG transporters may have a broad substrate specificity for a variety of structurally diversified substrates, and the substrate recognition specificity is probably determined by the specificity of dimer combinations (McFarlane *et al*., 2010). These studies promoted us to speculate LA5 may form different combinations of dimers to transport different substrates, which may contribute to its functional pleiotropy.

### LA5 positively regulates rice tiller angle by modulating LAT

Rice tiller angle is a complex trait with remarkable developmental plasticity, which is affected by environmental factors and endogenous factors such as auxin (Gao *et al*., 2019). In this study, we identified a novel factor *LA5*/*RCN1*/*OsABCG5* that regulates rice tiller angle through LAT. *LA5* overexpression leads to an larger tiller angle, while *LA5* RNAi or knockout results in a compact rice architecture (Figure 3b,c, Figure S4). Consistently, shoot gravitropism is decreased in *LA5* overexpression transgenic lines while increased in *LA5* RNAi transgenic lines (Figure S3b,c). Further evidence showed that lateral auxin transport is impaired in the *la5‐D* mutant (Figure 2b), and consequently, the asymmetric auxin distribution‐induced signal transduction is also attenuated (Figure 2c–e). These results demonstrated that *LA5* regulates rice tiller angle through modulating LAT.

Furthermore, we found LA5 interacts with OsPIN3t, which belongs to the PIN‐FORMED (PIN) protein family of auxin transporters (Figure 5a,b). Asymmetric auxin distribution‐induced signal transduction is attenuated in *OsPIN3t* overexpression transgenic lines (Figure S7b–d), indicating a negative role of *OsPIN3t* in regulating shoot LAT in rice, which is further supported by increased tiller angle in *OsPIN3t* overexpression transgenic lines (Figure 5d). Interestingly, *AtPIN3* was reported to positively regulate shoot LAT upon gravitropic stimulus in *Arabidopsis* (Friml *et al*., 2002). Although the detailed behaviour and function of OsPIN3t during shoot gravitropic response are largely unknown, it is likely that the regulatory manners of AtPIN3 and OsPIN3t regulate shoot gravitropism in a different manner during the divergence between *Arabidopsis* and rice.

Previous studies found the interaction between ABCB transporters and PIN proteins, such as the ABCB19‐PIN1 module (Blakeslee *et al*., 2007). The ABCB–PIN interactions appear to enhance auxin efflux activity, and function to motivate long‐distance auxin transport from the shoot to root apex (Mellor *et al*., 2022). Here, we found that the ABC transporter G subfamily member LA5/ABCG5 can interact with auxin transporter OsPIN3t to regulate asymmetric auxin transport (Figures 5a,b and 7). Considering that PAT and LAT, in which auxin moves directionally, were both impaired in the shoot of *LA5* overexpression transgenic lines, it would be very informative to investigate the effects of LA5 and OsPIN3t on auxin efflux. Further subcellular localization analysis *in planta* and auxin efflux activity characterization will illustrate the roles of LA5 and OsPIN3t in auxin transport. Our studies showed that the ABC transport G subfamily member can interact with the auxin transporter PIN protein, suggesting a novel potential regulatory mechanism of auxin transport in plant development.

### LA5 is a novel factor acting in the *LA1* ‐independent gravity signalling pathway controlling rice tiller angle

Previous study revealed that *LA1* null mutant exhibited only partially reduced gravitropism and incompletely prostrate growth (Li *et al*., 2007; Yoshihara and Iino, 2007). However, the partial defective shoot gravitropism of *la1* cannot be attributed to gene redundancy since *LA1* has no paralogue in rice (Li *et al*., 2007; Yoshihara and Iino, 2007). Therefore, previous study proposed that there may be an unidentified *LA1*‐independent pathway in regulating shoot gravitropism and rice tiller angle. A dozen of genes were found to regulate shoot gravitropism in *LA1*‐dependent pathway. However, few genes have been identified to act in the novel *LA1*‐independent pathway so far.

The Cholodny–Went model shows that auxin transport plays a central role in gravitropism by inducing differential growth (Firn *et al*., 2000). Lateral relocation of auxin efflux regulator PIN3 mediates gravitropism by relocating auxin flux laterally to determine differential growth (Friml *et al*., 2002; Kleine‐Vehn *et al*., 2010; Rakusova *et al*., 2011). Here, we identified a new component LA5 that negatively regulates rice LAT (Figures 2b and 7). PAT and LAT were both impaired in *la5‐D* (Figure 2a,b), while LAT was decreased but PAT was increased in *la1*, indicating *LA1* and *LA5* may regulate auxin transport differently. Moreover, LA5 interacts with OsPIN3t to regulate LAT, while LA1 interacts with BRXL4 to control LAT in rice shoots (Li *et al*., 2019), further suggesting the distinct functions of LA1 and LA5 in regulating rice shoot LAT (Figure 7). Distinguished from *la1* or *la5‐D* with partially defective LAT, LAT was almost abolished in *la1 la5‐D* (Figure 4f). Correspondingly, *LA5* overexpression further decreased the gravitropic response in *la1* and increased the tiller angle of *la1*, leading to near‐horizontal growth (Figure 4a–d, Figure S6a,b). These results demonstrated that *LA5* acts in a parallel genetic pathway independent of *LA1* controlling LAT. Therefore, we characterized a novel factor acting in a novel *LA1*‐independent gravity signalling pathway controlling rice tiller angle.

Crop architecture with ideal tiller angle and tiller number are strongly favoured in modern crop breeding programs. In this study, we demonstrated that *LA5* is important for the regulation of both tiller angle and tiller number, and up‐regulation of *LA5* not only enlarges tiller angle but also increases tiller number in rice. The discovery of *LA5* as an *LA1*‐independent regulator of tiller angle opens new avenues for precision breeding in rice. By modulating *LA5* expression or selecting natural haplotypes, breeders can improve rice architecture for high‐density farming and higher population yield. Future work should explore field performance of *LA5*‐edited lines to maximize yield grains. Our finding provides a strategy to balance tiller angle and tiller number for high‐yield breeding by appropriately modulating the expression of *LA5*.

## Methods

### Plant materials

The *OsPIN3t* overexpression (*OsPIN3t OE*) transgenic plants and the *la5‐D* mutant were in the background of ZH11 (*Oryza sativa* L. subsp. *japonica*), and the *CR‐la5* and *CR‐pin3t* mutants generated by CRISPR‐Cas9 technology in the background of ZH11. The *LA5 OE* and *RNAi* transgenic lines were in the background of Nipponbare (*Oryza sativa* L. subsp. *japonica*). The mutant of *la1* was reported in our previous study (Wang *et al*., 2024). The *OsPIN3t* overexpression (*OsPIN3t OE*) transgenic lines were described in previous study (Zhang *et al*., 2012). The *la1 la5‐D* and *la5‐D OsPIN3t OE‐1* were generated by crossing *la5‐D* with *la1* or *OsPIN3t OE‐1*, respectively. For field experiments, the rice plants were grown in Tai'an, Shandong province, in summer or in Lingshui, Hainan province, in winter, and the plants were spaced 30 cm apart and grown in paddy fields under natural conditions. Seedlings were grown on 0.4% agar plates at 28 °C under a 16‐h light/8‐h dark cycle for shoot gravitropism analysis.

### Map‐based cloning of *LA5*

To isolate *LA5*, we carried out map‐based cloning. The *la5‐D* mutant was crossed to the *indica* variety 93–11 (*Oryza sativa* L. subsp. *indica*) to produce the F_2_ mapping population. The In‐Del markers were generated based on nucleotide polymorphisms between the genome sequences of Nipponbare and 93–11. The primer sequences are listed in Table S1.

### Analysis of coleoptile gravitropism and shoot gravitropism

The gravitropic assay was carried out according to the method described previously (Li *et al*., 2007). The coleoptiles were grown at 28 °C for 3 days in darkness. The gravity response was determined by measuring the curved angle after reorienting by 90° for 4 h. Seedlings were grown at 28 °C under a 16‐h light/8‐h dark cycle. The gravity response was determined by measuring the curved angle after reorienting by 90° for a series of time periods in the dark or under a 16‐h light/8‐h dark cycle. For the gravitropism analysis of adult rice plants, plants were horizontally placed at heading stage for 2 weeks and the culm curvature was determined. To examine gene expression upon gravity stimulation by RT‐qPCR analyses, the assay was carried out according to the method described previously (Sang *et al*., 2014). Briefly, 7‐day‐old light grown seedlings were reoriented by 90° for 6 h, and then, 1.5 cm of the basal shoot was dissected into lower and upper sides.

### Vector construction and rice transformation

To generate the overexpression vector for *LA5*, the fragment containing the entire open reading frame of *LA5* were amplified and cloned into the binary vector pTCK303. The *CRISPR‐Cas9‐LA5* and *CRISPR‐Cas9‐OsPIN3t* vectors were constructed according to Cas9/gRNA kit (No. VK005‐01) (Viewsolid Biotech, Beijing, China). Above plasmids were introduced into *Agrobacterium tumefaciens EHA105* and transformed into ZH11 or Nipponbare was transformed as previously reported (Hiei *et al*., 1994). For generation of *LA5* RNAi transgenic plants in Nipponbare background, two fragments (121‐bp) of *LA5* were amplified from Nipponbare cDNA with two pairs of primers. The purified PCR products were ligated to the binary vector pTCK303. The probe plasmids TGMT‐Easy‐*LA5* (400 bp) were constructed for mRNA *in situ* hybridization. The primers are listed in Table S1.

### Real‐time PCR assays

Total RNA was extracted from various samples using TRIzol RNA extraction kit (Invitrogen, Carlsbad, CA) reagent according to the user manual. One microgram of total RNA was treated with DNase I and used to synthesize cDNA with an Avian Myeloblastosis Virus Reverse Transcriptase (Promega). Quantitative RT‐PCR (RT‐qPCR) experiments were performed using the SsoFast EvaGreen Supermix kit (Bio‐Rad) on the CFX96 real‐time system (Bio‐Rad, Hercules, CA) following the manufacturer's instructions. The expression levels were normalized to the expression of a rice ubiquitin gene. The primers were listed in Table S1.

### Polar auxin transport (PAT)

Polar auxin transport and lateral auxin transport were assayed as described previously with some modification (Li *et al*., 2007). Briefly, 5‐day‐old dark‐grown coleoptiles were removed from internally wrapped leaves, and apical sections (2 cm) were harvested and deprived of endogenous IAA. The apical or the basal ends of the segments (for basipetal or acropetal transport assays, respectively) were then vertically submerged into agar blocks containing 100 nM ^3^H‐IAA. 30 μM N‐1‐naphthylphtalamic acid (NPA) was added to the agar blocks in the control assay. After transport in darkness at 28 °C for 2.5 h, 5 mm sections from the non‐submerged ends of segments were excised and washed two times with 1/2 MS liquid medium. Five groups of coleoptile segments (5 mm) were used for the assay. After 24 h incubation in 400 μL scintillation liquid medium, the radioactivity of each section was counted by a liquid scintillation counter (1450MicroBeta TriLux, Pekin‐Elmer).

### Lateral auxin transport (LAT)

In the assay of lateral auxin transport, coleoptiles (1 cm) were harvested and deprived of endogenous IAA as mentioned above. The apical ends of coleoptiles were submerged into agar blocks that contain 100 nM ^3^H‐IAA. ^3^H‐benzoic acid was applied as a control. Segments from the apex were split evenly into upper and lower halves after transport in darkness at 28°C for 2.5 h. After 24 h incubation in 400 μL scintillation liquid, the radioactivity of each half was counted as described above.

### *In situ* hybridization

RNA *in situ* hybridization was performed according to a previous report with minor modification (Xu *et al*., 2012). Shoot bases of 14‐day‐old rice seedlings were put into the vacuum tissue processor (ASP200, Leica) to fix, dehydrate, clear and embed and were subsequently embedded in paraffin (Paraplast Plus, Sigma). The shoot bases were sliced into 10‐μm sections with a microtome (Leica RM2145). The *LA5* cDNA was amplified with primer pairs TGMT Easy‐LA5‐TZ400‐F/TGMT Easy‐LA5‐TZ400‐R and subcloned into the TA Cloning vector (Table S1), which was used as the template to generate sense and antisense RNA probes. Digoxigenin‐labelled RNA probes were prepared using a DIG Northern Starter Kit (Roche) according to the manufacturer's instruction. Slides were observed under bright field through a microscope (Leica DMR) and photographed with a micro‐colour charge‐coupled device (CCD) camera (Apogee Instruments).

### Yeast two‐hybrid assays

For yeast two‐hybrid screening, the full‐length open reading frame of *LA5* was amplified and subcloned into pDHB1 bait vectors of the Dualhybrid system as bait. cDNA libraries from rice seedlings were introduced to pPR3‐N prey vectors. The combinatory constructs were transformed simultaneously into the NMY51 yeast strain and tested for Leu, Trp, His and Ade auxotrophs. LacZ (ß‐galactosidase assay) reporter activity was monitored using the HTX β‐galactosidase assay kit (DualsystemsBiotech) according to the manufacturer's protocols.

### Luciferase complementation imaging (LCI) assay

To generate the constructs of *35S*_*pro*_ *:LA5‐nLUC* and *35S*_*pro*_ *:OsPIN3t‐cLUC*, the full‐length CDSs of *LA5* and *OsPIN3t* without stop codons were amplified from ZH11 cDNA and inserted into *35S*_*pro*_ *:nLUC* and *35S*_*pro*_ *:cLUC* vectors, respectively. The nLUC and cLUC fusion vectors were transformed into *A. tumefaciens* EHA105 and then co‐injected into *N. benthamiana* leaves. The luciferase complementation was observed by NightShade LB 985 In Vivo Plant Imaging System (IndiGO; Berthold Technologies Co., Bad Wildbad, Germany) using beetle luciferin as the substrate (Promega, E1603). Primers used for the LCI assay are listed in Table S1.

### Haplotype and selective signal analysis of *LA5*

We used the high‐density SNP set of rice based on high‐density whole genome resequencing data of both wild and cultivated rice germplasms (acquired from National Genomics Data Center, China National Center for Bioinformation with accession GVM000285) for the haplotype and selective signal analysis of *LA5*‐related regions. Haplotype blocks were calculated using Plink v1.90b6.13 with options ‘‐ld‐window‐kb 500‐blocks‐min‐maf 0.05‐blocks‐strong‐lowci 0.7‐blocks‐strong‐highci 0.98’. Variants in the haplotype blocks that containing the flanking 2 kb region of *LA5* gene were extracted for downstream analyses. For haplotype clustering, homozygous reference, homozygous alternative and heterozygous genotypes were recoded to −1, 0, 1, respectively. The pairwise euclidean distances were then calculated based on the recoded genotypes, and the hierarchical cluster analysis was performed (hclust in R version 4.0.0, with method ‘complete’), and the number of clusters was determined based on the silhouette analysis of kmeans clustering using R/factoextra. The haplotype network analysis was performed based on the whole genotype matrix of the haplotype block (kept the variants with heterozygous genotypes or missing genotypes) using random minimum span tree embedded in R/pegas. The nucleotide diversities of different rice populations were calculated using vcftools version0.1.16 with options ‘‐‐window‐pi 50000 ‐‐window‐pi‐step 1000’.

## Conflict of interest

The authors declare that they have no conflicts of interest.

## Author contributions

W.W., D.S. and P.L. designed research, performed experiments and analysed data; W.W. and D.S. wrote the manuscript; S.G., Y.L., Y.S., H.Z., J.C., Z.W., X.M. performed some of the experiments; J.L. and H.X. analysed the data; Y.W. supervised the project, designed research, analysed data and wrote the manuscript. W.W., D.S. and P.L. contributed equally to this work.

## Supporting information


**Table S1** Primers used in this study.

**Figure S1** Shoot gravitropism analysis of the wild type ZH11 and *la5‐D*.
**Figure S2** Characterization of the T‐DNA insertion in the mutant *la5‐D*.
**Figure S3** Shoot gravitropism and tiller number of the *LA5* transgenic plants upon gravistimulation.
**Figure S4** Generation and phenotype analysis of *CR‐la5* transgenic plants.
**Figure S5** Expression pattern analysis of *LA5* by using qRT‐PCR.
**Figure S6** Shoot gravitropism analysis of the *la1 la5‐D* mutant.
**Figure S7** Expression of *OsPIN3t* in the rice *OsPIN3t OE* transgenic plants and the expression of auxin response genes upon gravistimulation.
**Figure S8** Geographic distribution of different haplotype clusters of *LA5*.

## Data Availability

The data that supports the findings of this study are available in the supplementary material of this article.
