# Supplementary material for: LAZY5 acts in an LAZY1‐independent pathway to regulate rice tiller angle
Source: Plant Biotechnol J. 2025 Jul 13;23(10):4568–79. doi: 10.1111/pbi.70211 (PMC12483965; doi:10.1111/pbi.70211)
Supplement: Supplementary file 1 — Table S1 Primers used in this study. [file PBI-23-4568-s001.docx]

**Supplemental Table 1**. Primers used in this study.

| Marker Primer sequences（5′-3′） |
| --- |
| M1-F AGACTCTACATCATAAGCCT  M1-R CTGTGCCCAATTACCCTAACCTC  M2-F CACATGATGCTCTTAGGTGACTT  M2-R CAGAGCAGTTTCCATGTGATACA  M3-F CGTGATGGGAGGCAGTTCA  M3-R TCTAGTGGTAGATCAGCGTCTGTAT  M4-F TCCAACTACACTCAACTCC  M4-R ACCATTTCCGACCAATCT  M5-F CCCCACGAATCTTAGCCTC  M5-R CATCATGGCTTCAGCTTCC  M6-F ATGAAGACTTTAATTTGGCACT  M6-R GACATCTACGGCGACAACAGC  M8-F ATAAACAAGGCTAACCGTCACC  M8-R GCTCTGCTGCTGACTTCCCT  M9-F AAGATGAGTGTTTGAAAGAGAATAGT  M9-R TTAATTTCTTGTCACATCATAAAAG  M10-F CGGTTTTGAACGTGCAGGA  M10-R AATCATGCACTAATAATTTACATCG  P-F GCCCCACATAATCTTGGAACA  P-R CAAAGTCAGGAAATGTGGAGGC  T-F TGCCTTGGTTTGTGAAGCAAGCC  pTCK303-LA5 -OE–F (*Kpn*1) AAGGTACCATGTCGCGGTTTGTCGACAA  pTCK303-LA5 -OE-R (*Sac*I) AAGAGCTCCTACCTCCGCTTGTTCCTGCT  pTCK303-LA5-RNAi–1F (*Bam*HI) AAGGATCCTACTACCAGCCGCACAGCAAC  pTCK303-LA5 -RNAi–1R (*Kpn*I) AAGGTACCACGACCCCTTCCTCCGCT  pTCK303-LA5 -RNAi–2F (*Sac*I) AAGAGCTCTACTACCAGCCGCACAGCAAC  pTCK303-LA5 -RNAi–2R (*Spe*I) AAACTAGTACGACCCCTTCCTCCGCT  TGMT Easy-LA5 -TZ400-F ATGTCGCGGTTTGTCGACAAGC  TGMT Easy-LA5-TZ400-R TCCGCTGCTTCACGCTGTAC  qRT-LA5-F GGGTTCTTCTACTTCGTGGCAAT  qRT-LA5- R ATGTAGTGGAACCACAGCCAGTAG  qWOX11_F CGGTGTTCATCAACGGAGTG  qWOX11_R TCTGGAGAGAATGGAGGAGGAT  qWOX6_F TCCAATAGACTTGCGAGCCAT  qWOX6_R GCATTAGGATTCCATAGTCGTT  qOsIAA20-F TGGCGGATATGTGAAGGTGAA  qOsIAA20-R TATGAGCCGAGGATGGACAAG  qLA5- F ACCATGTACTACACCTGCTCCG  qLA5-R TTGTACGCCGTCTCCCTGA  qUbi-F AACCAGCTGAGGCCCAAGA  qUbi-R ACGATTGATTTAACCAGTCCATGA  CRISPR-*LA5* -1F CAGGTGCCTGCCGTTCCGTCGTG  CRISPR-*LA5* -1R AACCACGACGGAACGGCAGGCAC CRISPR-*LA5* -2F CAG GCTCGACGGCATCTCCGGCG  CRISPR-*LA5* -2R AAC CGCCGGAGATGCCGTCGAGC  CRISPR-OsPIN3t-1F CAGTCGCGCCTCCCCTCGCGGAC  CRIPSR-OsPIN3t-1R AACGTCCGCGAGGGGAGGCGCGA  CRISPR-OsPIN3t-2F CAGGCCATGTACGGGCCATACTC  CRIPSR-OsPIN3t-2R AACGAGTATGGCCCGTACATGGC |
