# Supplementary material for: LAZY5 acts in an LAZY1‐independent pathway to regulate rice tiller angle
Source: Plant Biotechnol J. 2025 Jul 13;23(10):4568–79. doi: 10.1111/pbi.70211 (PMC12483965; doi:10.1111/pbi.70211)
Supplement: Supplementary file 2 — Figure S1 Shoot gravitropism analysis of the wild type ZH11 and la5‐D. Figure S2 Characterization of the T‐DNA insertion in the mutant la5‐D. Figure S3 Shoot gravitropism and tiller number of the LA5 transgenic plants upon gravistimulation. Figure S4 Generation and phenotype analysis of CR‐la5 transgenic plants. Figure S5 Expression pattern analysis of LA5 by using qRT‐PCR. Figure S6 Shoot gravitropism analysis of the la1 la5‐D mutant. Figure S7 Expression of OsPIN3t in the rice OsPIN3t OE transgenic plants and the expression of auxin response genes upon gravistimulation. Figure S8 Geographic distribution of different haplotype clusters of LA5. [file PBI-23-4568-s002.pdf]

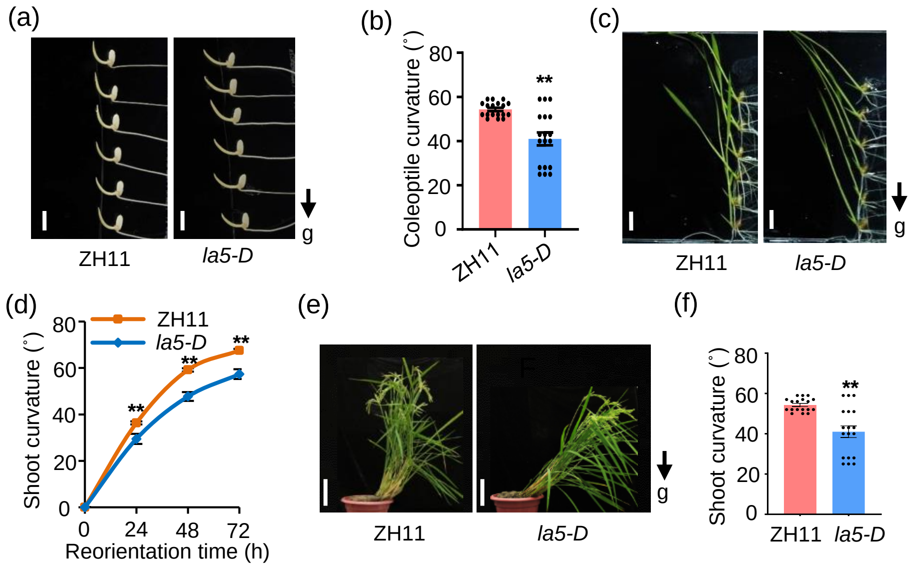

Figure S1 Shoot gravitropism analysis of the wild type ZH11 and *la5-D*.

(a) Coleoptile curvature of ZH11 and *la5-D* after gravistimulation for 4 h. The arrow indicates the direction of gravity. Scale bars, 1cm. (b) Statistical analysis of coleoptiles curvature grown under dark after gravity stimulation for 4 h. Data are presented as mean ± SE (n = 18). **P < 0.01, Student's t-test. (c) Phenotypes of seedlings grown under light after gravistimulation for 72 h in the dark. The black arrow indicates the direction of gravity. Scale bars, 1cm. (d) Statistical analysis of shoot curvature grown under light after gravistimulation in the dark. Data are presented as mean ± SE (n = 16). **P < 0.01, Student's t-test. (e) The phenotype of the wild type and *la5-D* at the adult stage after gravistimulation. The black arrow indicates the direction of gravity. Scale bars, 10 cm.

(f) Statistical analysis of the node II curvature of WT and *la5-D* at the adult stage after gravistimulation. Data are presented as mean ± SE (n = 15). **P < 0.01, Student's t-test.

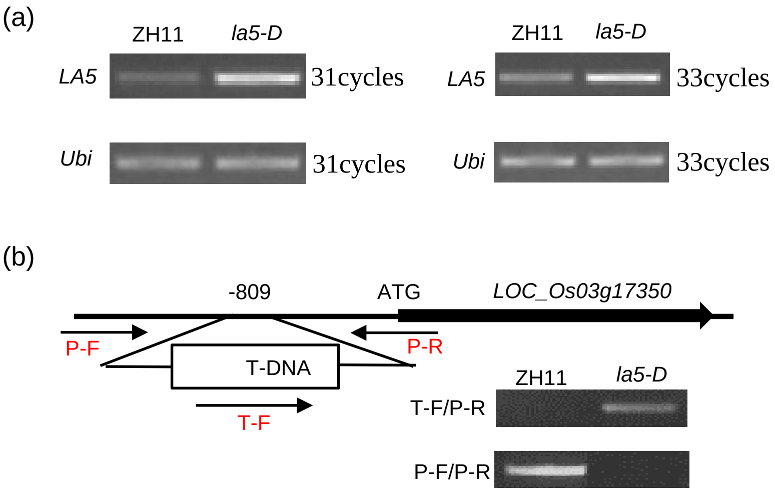

(c)

ATCCTTGGAGCTGCTTATTTTAGTACCAGTGTGTTTGTTGGCTTTTTTTTTAGCGTGTTTGTTTCTGCAGC
TTTGGAGATGCAACACCATTGTCAATGCTTTTGTTGGTTGGAGAAACTGTTCCGATGAGCAAATGGATA
AGTTTTGGCTTCCCTTGCAGAGATGAATTGTTTTATGCTTTAATTC**A**ATTGTAAATCTCGAAGTCGACGG
ATCCGGTACCGTTAACTCGAGATCTAGATATCGATGAATTCGAGCTCGGTACCCCCCGGGAATTCCCTTT
CAGAAAGAATGCTAACCCACAGATGGTTAGAGAGGCTTACGCAGCAGGTCTCATCAAGACGATCTACC
CGAGCAATAATCTCCAGGAAATCAAATACCTTCCCAAGAAGGTTAAAGATGCAGTCAAAAGATTCAGG
ACTAACTGCATCAAGAACACAGAGAAAGATATATTTCTCAAGATCAGAAGTACTATTCCAGTATGGACG
ATTCAAGGCTTGCTTCACAAACCAAGGCAAGTAATAGAGATTGGAGTCTCTAAAAAGGTAGTTCCCAC
TGAATCAAAGGCCATGGAGTCAAAGATTCAAATAGAGGACCTAACAGAACTCGCCGTAAAGACTGGC
GAACAGTTCATACAGAGTCTCTTACGACTCAATGACAAGAAGAAAATCTTCGTCAACATGGTGGAGCA
CGACACACTTGTCTACTCCAAAAATATCAAAGATACAGTCTCAGAAGACCAAAGGGCAATTTGAGACTT
TTTCAACAAAGGGTAATATCCCGGAAACCTCCTCGGATTCCATTTGCCCAGCTATCTGTCACTTTTATTGT
GAAGATAGTGGAAAGGAAGGTGGCTCCTACAAATGCCATCATTGCGATAAAGGAAAGGCATCGTTGA
AGATGCTCTGCCGACGTGTGCCAAGATGGACCCCTACCCCACGAGAGCTCGTGAACATGAGACGTGCC
TATCCACGTCCTCAAG

Figure S2 Characterization of the T-DNA insertion in the mutant *la5-D*. (a) Expression level of *LA5* in the wild type ZH11 and *la5-D*. (b) T-DNA insertion was confirmed by the genomic PCR. P-F, P-R and T-F are the identification primers of the T-DNA insertion. (c) Sequencing analysis of the T-DNA insertion position. Yellow colored position indicates where T-DNA is inserted.

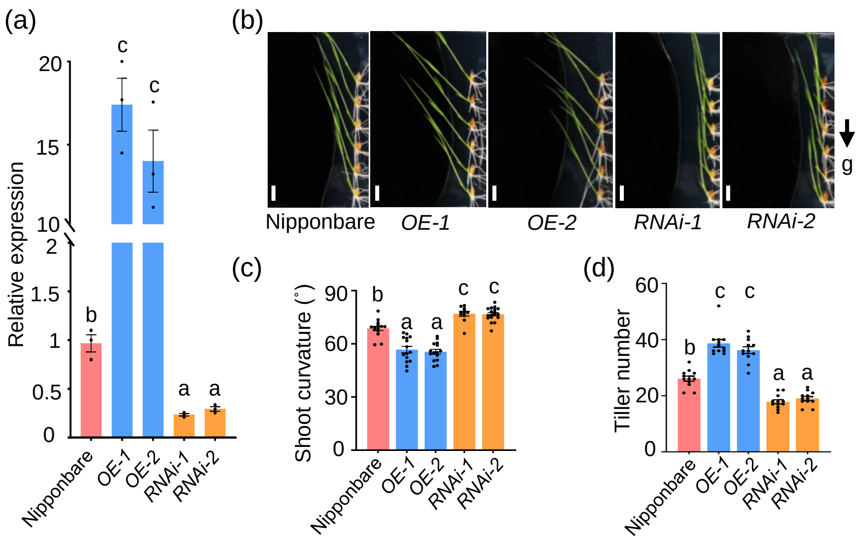

Figure S3 Shoot gravitropism and tiller number of the *LA5* transgenic plants upon gravistimulation.

(a) Expression levels of *LA5* in the overexpressing and RNAi transgenic plants of *LA5*. Data are presented as mean ± SE (n = 3). (b) Phenotypes of seedlings grown under the light after gravistimulation for 72 h in the dark. The black arrow indicates the direction of gravity. Scale bars, 1cm. (c) Statistical analysis of shoot curvature grown under the light after gravity stimulation in the dark. Data are presented as mean ± SE (n = 16). Different letters above the column represent statistically significant difference at $P < 0.05$ (one-way ANOVA, Tukey's honestly significant difference). (d) Statistical analysis of the tiller number of the overexpression and RNAi transgenic plants of *LA5*. Data are presented as mean ± SE (n = 10). **$P < 0.01$, Student's t-test. Different letters above the column represent statistically significant difference at $P < 0.05$ (one-way ANOVA, Tukey's honestly significant difference).

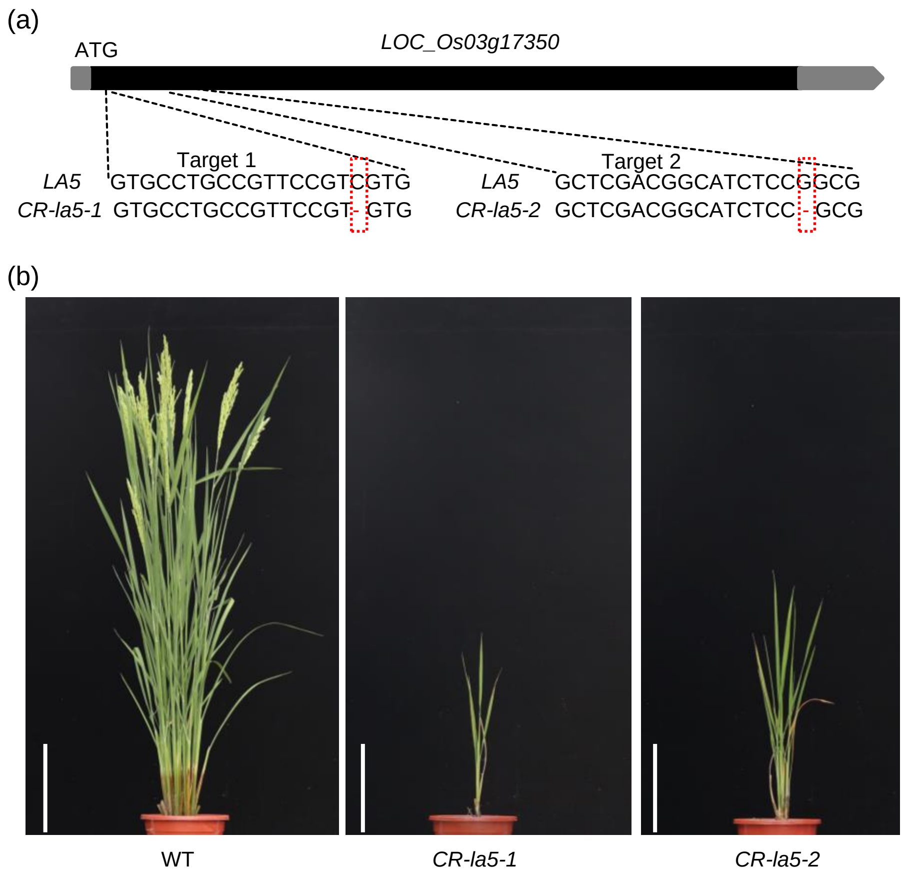

Figure S4 Generation and phenotype analysis of *CR-la5* transgenic plants.
(a) Schematic illustration of the two gRNA target sites of *LA5* in the CRISPR-Cas9 engineered *la5* mutant lines. The black boxes represent coding region of *LA5* while the gray ones indicate untranslated regions. The sgRNA target sequences are listed below. The '–' in the red box represents the 1-bp deletion in the target sites of the loss-of-function mutant *la5* mutants. (b) Phenotypes of the loss-of-function mutant *la5*. Scale bars, 25 cm.

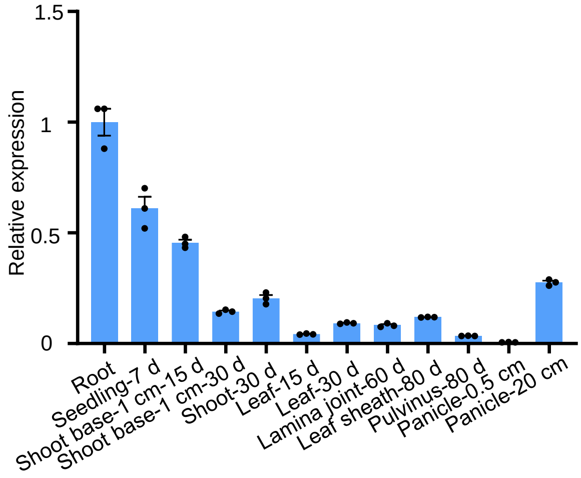

Figure S5 Expression pattern analysis of *LA5* by using qRT-PCR.
Expression pattern of *LA5* in different rice organs. Data are represented as mean ± SEM (*n* = 3).

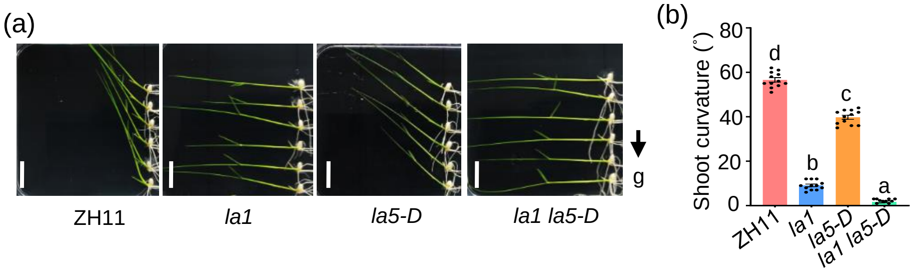

Figure S6. Shoot gravitropism analysis of the *la1 la5-D* mutant.

(a) Seedlings gravitropism of *la1 la5-D* mutant. g: gravity. Black arrow indicates the direction of gravistimulation. Scale bars, 2cm. (b) Statistical analysis of the shoot curvature angle of the *la1 la5-D* mutant. Data are represented as mean ± SE (n = 12). Different letters above the column represent statistically significant different at P<0.05 (One-way ANOVA. Tukey`s honestly significant difference).

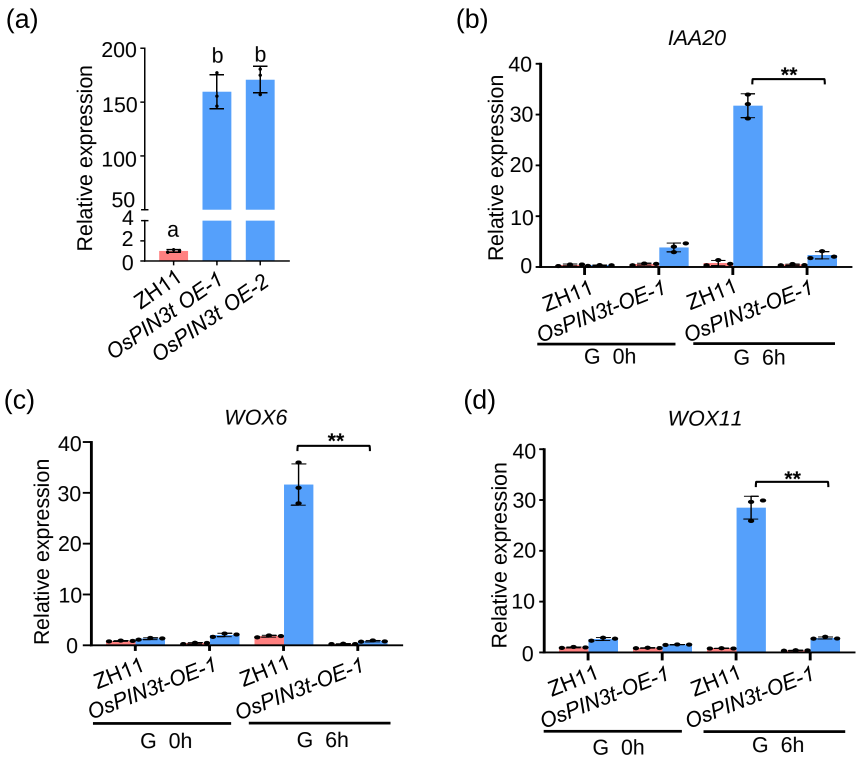

Figure S7 Expression of *OsPIN3t* in the rice *OsPIN3t OE* transgenic plants and the expression of auxin response genes upon gravistimulation.

(a) Expression levels of *OsPIN3t* in the *OsPIN3t* overexpression transgenic plants. Data are presented as mean±SE (n = 3). Different letters above the column represent statistically significant different at P<0.05 (One-way ANOVA. Tukey`s honestly significant difference).

(b-d) Expression levels of *IAA20* (b), *WOX6* (c), and *WOX11* (d) in the lower side and the upper side of shoot bases of young seedlings upon gravistimulation for 0 h and 6 h, respectively. Data are presented as mean ± SE (n = 3). **P < 0.01, Student's t-test.

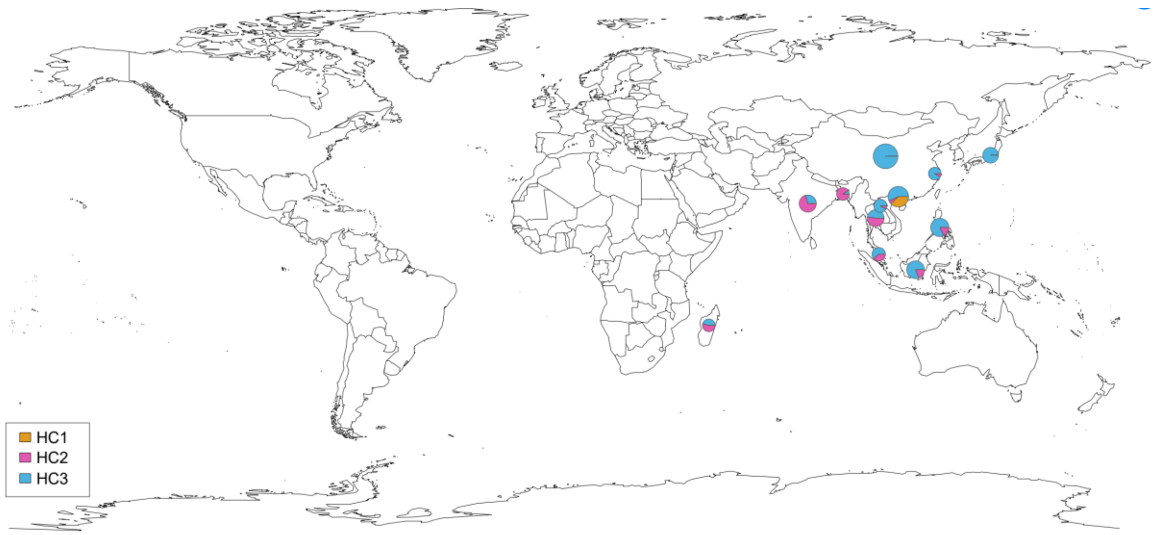

Figure S8 Geographic distribution of different haplotype clusters of *LA5*.
HC1~HC3: different haplotype clusters as illustrated in Figure 6A. *O.rufipogon* (HC1), *O.sativa*.ssp.*indica* (HC2) and *O.sativa*.ssp.*japonica* (HC3).
